# Concurrence of water and food insecurities, 25 low- and middle-income countries

**DOI:** 10.2471/BLT.22.288771

**Published:** 2022-12-01

**Authors:** Sera L Young, Hilary J Bethancourt, Edward A Frongillo, Sara Viviani, Carlo Cafiero

**Affiliations:** a Department of Anthropology, Northwestern University, 1819 Hinman Avenue, Evanston, Illinois, 60201 United States of America (USA).; b Department of Health Promotion, Education, and Behavior, University of South Carolina, Columbia, USA.; c Statistics Division, Food and Agriculture Organization of the United Nations, Rome, Italy.

## Abstract

**Objective:**

To investigate how water and food insecurity were associated in nationally representative samples of individuals from 25 low- and middle-income countries.

**Methods:**

We used data from the 2020 World Gallup Poll in which the Individual Water Insecurity Experiences Scale and the Food Insecurity Experience Scale had been administered to 31 755 respondents. These scales measure insecurity experiences in the previous 12 months. We classified individuals as water insecure if their score was ≥ 12 and food insecure if the Rasch probability parameter was ≥ 0.5. For estimating the proportions, we used projection weights. We estimated the relationships between binary and continuous measures of water insecurity and food insecurity for individuals within each country and region using multivariable logistic and linear regression models, adjusting for key socioeconomic characteristics including income, gender, age and education.

**Findings:**

Among the 18.3% of respondents who experienced water insecurity, 66.8% also experienced food insecurity. The likelihood of experiencing moderate-to-severe food insecurity was higher among respondents also experiencing water insecurity (adjusted odds ratio, aOR: 2.69; 95% confidence interval, CI: 2.43 to 2.98). Similar odds were found in Asia (aOR: 2.95; 95% CI: 2.04 to 4.25), Latin America (aOR: 2.17; 95% CI: 1.62 to 2.89), North Africa (aOR: 2.92; 95% CI: 2.17 to 3.93) and sub-Saharan Africa (aOR: 2.71; 95% CI: 2.40 to 3.06).

**Conclusion:**

Our results suggest that water insecurity should be considered when developing food and nutrition policies and interventions. However, more research is needed to understand the paths between these insecurities.

## Résumé

**Objectif:**

Analyser la manière dont l'insécurité alimentaire et l'insécurité liée à l'approvisionnement en eau se combinent au sein d'échantillons d'individus représentatifs à l'échelle nationale dans 25 pays à revenu faible et intermédiaire.

**Méthodes:**

Nous avons utilisé les données issues du Sondage mondial Gallup réalisé en 2020, qui avait soumis l'Échelle de l'insécurité hydrique (WIES) et l'Échelle de l'insécurité alimentaire (FIES) à 31 755 participants. Ces échelles mesurent le sentiment d'insécurité éprouvé au cours des 12 mois écoulés. Nous avons considéré que les personnes souffraient d'insécurité hydrique lorsque leur score était ≥ 12, et d'insécurité alimentaire lorsque le paramètre de probabilité du modèle de Rasch était ≥ 0,5. Pour estimer les proportions, nous avons employé des poids prévisionnels. Enfin, nous avons établi des liens entre les mesures binaires et continues de l'insécurité hydrique et alimentaire pour les habitants de chaque pays et région à l'aide de modèles de régression linéaire et logistique multivariée, ajustés en fonction de plusieurs caractéristiques socioéconomiques clés telles que le revenu, le genre, l'âge et le niveau d'éducation.

**Résultats:**

Parmi les 18,3% participants touchés par l'insécurité hydrique, 66,8% souffraient également d'insécurité alimentaire. Les participants sujets à l'insécurité hydrique étaient également davantage susceptibles d'être confrontés à une insécurité alimentaire modérée à sévère (odds ratio ajusté, ORA: 2,69; intervalle de confiance de 95%, IC: 2,43 à 2,98). Les probabilités étaient similaires en Asie (ORA: 2,95; IC de 95%: 2,04 à 4,25), en Amérique latine (ORA: 2,17; IC de 95%: 1,62 à 2,89), en Afrique du Nord (ORA: 2,92; IC de 95%: 2,17 à 3,93) et en Afrique subsaharienne (ORA: 2,71; IC de 95%: 2,40 à 3,06).

**Conclusion:**

Nos résultats suggèrent que l'insécurité liée à l'approvisionnement en eau devrait être prise en compte dans l'élaboration d'interventions et de politiques relatives à l'alimentation et à la nutrition. Cependant, d'autres recherches sont nécessaires pour mieux comprendre les liens qui unissent ces deux types d'insécurité.

## Resumen

**Objetivo:**

Investigar cómo se asocia la inseguridad alimentaria y el agua en muestras representativas a nivel nacional de personas de 25 países de ingresos bajos y medios.

**Métodos:**

Se utilizaron datos de la Encuesta Mundial Gallup 2020 que había administrado la Escala de Experiencias de Inseguridad Hídrica Individual y la Escala de Experiencias de Inseguridad Alimentaria entre 31 755 encuestados. Estas escalas miden las experiencias de inseguridad en los 12 meses anteriores. Se clasificó a las personas como inseguras con respecto al agua si su puntuación era ≥12 y como inseguras con respecto a los alimentos si el parámetro de probabilidad de Rasch era ≥0,5. Para estimar los porcentajes, se utilizaron pesos de proyección. Se estimaron las relaciones entre las medidas binarias y continuas de inseguridad hídrica e inseguridad alimentaria para las personas dentro de cada país y región utilizando modelos multivariables de regresión logística y lineal, ajustando por características socioeconómicas clave, incluidos los ingresos, el género, la edad y la educación.

**Resultados:**

Entre el 18,3 % de los encuestados que experimentaron inseguridad hídrica, el 66,8 % también experimentó inseguridad alimentaria. La probabilidad de sufrir inseguridad alimentaria de moderada a grave era mayor entre los encuestados que también sufrían inseguridad hídrica (razón de posibilidades ajustada, RPA: 2,69; intervalo de confianza del 95 %, CI: 2,43 a 2,98). Se encontraron posibilidades similares en Asia (RPA: 2,95; IC del 95 %: 2,04 a 4,25), América Latina (RPA: 2,17; IC del 95 %: 1,62 a 2,89), África del Norte (RPA: 2,92; IC del 95 %: 2,17 a 3,93) y África subsahariana (RPA: 2,71; IC del 95 %: 2,40 a 3,06).

**Conclusión:**

Los resultados sugieren que la inseguridad hídrica se debería tener en cuenta al elaborar políticas e intervenciones en materia de alimentación y nutrición. Sin embargo, se necesita más investigación para comprender los vínculos entre estos dos tipos de inseguridad.

## ملخص

الغرض التحقيق في كيفية الارتباط بين انعدام الأمن المائي وانعدام الأمن الغذائي في عينات تمثيلية على المستوى الوطني لأفراد من 25 دولة منخفضة الدخل ومتوسطة الدخل.

**الطريقة:**

قمنا بالاستعانة ببيانات من استطلاع World Gallup Poll لعام 2020، الذي سعى لتقييم مقياس تجارب انعدام الأمن المائي الفردي، ومقياس تجربة انعدام الأمن الغذائي الفردي بين 31755 مشاركًا. وتقيس هذه المقاييس تجارب انعدام الأمن في الـ 12 شهرًا الماضية. قمنا بتصنيف الأفراد على أنهم يعانون من انعدام الأمن المائي إذا كانت درجاتهم أكبر من أو تساوي 12، وأنهم يعانون من انعدام الأمن الغذائي إذا كان معامل احتمالية Rasch أكبر من أو يساوي 0.5. لتقدير النسب، قمنا باستخدام أوزان الإسقاط. قمنا بتقدير العلاقات بين المقاييس الثنائية والمستمرة لانعدام الأمن المائي وانعدام الأمن الغذائي للأفراد داخل كل بلد ومنطقة، باستخدام نماذج التحوّف الخطي واللوجستي متعدد المتغيرات، مع تعديل الخصائص الاجتماعية والاقتصادية الرئيسية بما في ذلك الدخل، والجنس، والعمر، والتعليم.

**النتائج:**

من بين 18.3% من المشاركين الذين عانوا من انعدام الأمن المائي، عانى 66.8% أيضًا من انعدام الأمن الغذائي. كان احتمال المعاناة من انعدام الأمن الغذائي المعتدل إلى الشديد أعلى بين المشاركين الذين عانوا أيضًا من انعدام الأمن المائي (نسبة الاحتمالات المعدلة: 2.69؛ بفاصل ثقة مقداره 95%: 2.43 إلى 2.98). تم العثور على احتمالات متشابهة في آسيا (نسبة الاحتمالات المعدلة: 2.95؛ ‏بفاصل ثقة مقداره 95%: 2.04 إلى 4.25)، وأمريكا اللاتينية (نسبة الاحتمالات المعدلة: 2.17؛ ‏بفاصل ثقة مقداره 95%: 1.62 إلى 2.89)، وشمال أفريقيا (نسبة الاحتمالات المعدلة: 2.92؛ ‏بفاصل ثقة مقداره 95%: 2. 17 إلى 3.93)، وجنوب الصحراء الكبرى (نسبة الاحتمالات المعدلة: 2.71؛ ‏بفاصل ثقة مقداره 95%: 2.40 إلى 3.06).

**الاستنتاج:**

تشير نتائجنا إلى أنه يجب وضع انعدام الأمن المائي في الاعتبار عند وضع سياسات وتدخلات الغذاء والتغذية. ومع ذلك، هناك حاجة إلى مزيد من البحث لفهم المسارات بين حالات عدم الأمان هذه.

## 摘要

**目的:**

旨在针对来自 25 个中低收入国家的具有国家代表性的个人，调查水和食物不安全问题是如何关联的。

**方法:**

我们使用了 2020 年盖洛普世界民意调查中的数据，该调查要求 31,755 名受访者填写《个人水不安全体验量表》和《食物不安全体验量表》，以衡量其过去 12 个月的不安全体验。我们将得分 ≥ 12 的受访者归入“水不安全”类别，并将 Rasch 概率参数 ≥ 0.5 的受访者归入“食物不安全”类别。为了估算比例，我们使用了投影权重。我们使用多变量逻辑回归和线性回归模型，估计了在每个国家和地区范围内个人水不安全性和食物不安全性的二元和连续测量结果之间的关系，并对包括收入、性别、年龄和教育在内的关键社会经济特征进行了调整。

**结果:**

在面临水不安全问题的 18.3% 受访者中，66.8% 同时面临食物不安全问题。这些同时面临水不安全问题的受访者遇到中等至严重食物不安全问题的可能性更大（调整后优势比，aOR：2.69；95％ 置信区间，CI：2.43 至 2.98）。在亚洲（aOR：2.95；95% CI：2.04 至 4.25）、拉丁美洲（aOR：2.17；95% CI：1.62 至 2.89）、北非（aOR：2.92；95% CI：2.17 至 3.93）和撒哈拉以南非洲（aOR：2.71；95% CI：2.40 至 3.06）都发现存在类似的可能性。

**结论:**

我们的结果表明，在制定食物和营养政策和干预措施时，应考虑水不安全问题。然而，同时需要开展更多研究以了解这些不安全问题之间的关系。

## Резюме

**Цель:**

Изучить, как связаны нехватка воды и продовольствия в репрезентативных выборках людей на национальном уровне из 25 стран с низким и средним уровнем дохода.

**Методы:**

Были использованы данные Всемирного опроса Гэллапа за 2020 год, в ходе которого 31 755 респондентам предоставлялись индивидуальная шкала восприятия отсутствия водной безопасности и шкала восприятия отсутствия продовольственной безопасности. По этим шкалам измеряется восприятие отсутствия безопасности в течение предыдущих 12 месяцев. Авторы считают, что у лиц c количеством баллов ≥ 12 наблюдалась нехватка воды, а при значении параметра вероятности по модели Раша ≥ 0,5 – нехватка продовольствия. Для оценки пропорций использовались проекционные весовые коэффициенты. Была проведена оценка взаимосвязи между бинарными и непрерывными показателями нехватки воды и продовольствия для отдельных лиц в каждой стране и регионе с использованием многомерных логистических и линейных регрессионных моделей с поправкой на основные социально-экономические характеристики, включая доход, пол, возраст и образование.

**Результаты:**

Из 18,3% респондентов, столкнувшихся с нехваткой воды, 66,8% также ощущали нехватку продовольствия. Вероятность отсутствия продовольственной безопасности от умеренной до тяжелой степени была выше среди респондентов, столкнувшихся также с нехваткой воды (скорректированное отношение шансов, сОШ: 2,69; 95%-й ДИ: 2,43–2,98). Аналогичные шансы были обнаружены в Азии (сОШ: 2,95; 95%-й ДИ: 2,04–4,25), Латинской Америке (сОШ: 2,17; 95%-й ДИ: 1,62–2,89), Северной Африке (сОШ: 2,92; 95%-й ДИ: 2,17–3,93) и Африке южнее Сахары (сОШ: 2,71; 95%-й ДИ: 2,40–3,06).

**Вывод:**

Полученные результаты свидетельствуют о том, что при разработке политики и мероприятий в области продовольствия и питания следует учитывать отсутствие водной безопасности. Однако необходимо провести дополнительные исследования, чтобы понять, как связаны между собой упомянутые выше нехватки.

## Introduction

Food insecurity, that is, lack of stable access to sufficient, safe and nutritious foods to meet nutritional needs,^1^ is widespread and has adverse effects on health and well-being.^2^ Mounting evidence shows that, regardless of household income, food security and nutrition are negatively affected by water insecurity, defined as lack of stable access of sufficient and safe amounts of water for drinking and domestic uses.^3^^–^^9^ For example, lack of water or poor-quality water could hinder food preparation, prevent people cooking more nutritious foods or cooking any food whatsoever,^8^^–^^17^ resulting in them going without eating or resorting to less nutritious foods (e.g. highly processed packaged foods) that require little or no water. Water shortages and flooding events may also prevent households and communities from growing their own food, from growing cash crops and/or from raising livestock for food or income.^7^^,^^10^^–^^12^^,^^14^^,^^18^ Furthermore, if water insecurity necessitates more money to be spent on water, fewer funds may be available for food purchases.^9^^,^^12^^,^^17^^,^^19^ Money to purchase food may also be compromised by limited water access if time required to fetch water off premises takes away time for income-generating tasks.^13^^,^^17^^,^^20^

The limited data available suggest that household water insecurity and household food insecurity tend to be positively associated. For example, researchers have shown positive associations between household food insecurity scores and time required to fetch water,^21^ perceived water cleanliness^14^ and experiential measures of water insecurity.^22^^–^^25^ Relationships between experiential water insecurity and food insecurity persisted when adjusting for perceived social status^9^ and water expenditures.^19^ Because water insecurity has been measured differently across these studies and often only assessed in bivariate analyses, it remains unclear where or in which contexts the potential impact of water insecurity on food insecurity is more severe when adjusting for common causes, like socioeconomic status.

A further limitation in our understanding of the relationships between water insecurity and food insecurity is that disparities by individual characteristics such as gender, age and health status have gone unmeasured. Knowing where and among which individuals water and food insecurities coexist, and whether this coexistence is independent of socioeconomic factors, could offer more precise insights than household-level indicators. These insights could be used for developing appropriately targeted interventions and policies to mitigate food insecurity that are not undermined by concurrent problems with water.^6^^,^^7^

Finally, most studies have been site-specific and not nationally representative. The few studies that have used nationally representative samples measured water in terms of access to an improved water source or distance between the home and water source.^26^^–^^29^ These studies did not measure if the available water sources provide sufficient and stable water for domestic uses, including food preparation, and hence made an incomplete measurement of water insecurity.

Therefore, we investigated how water access, use and stability related to individual food insecurity in 25 low- and middle-income countries, using a national representative survey. We sought to (i) describe the extent to which water insecurity and food insecurity were concurrently experienced in these countries; (ii) test if the likelihood of being moderately to severely food insecure was higher among those who were water insecure when controlling for socioeconomic confounders by both country and region; and (iii) test if the severity of water insecurity predicted the severity of food insecurity when controlling for socioeconomic confounders.

## Methods

### Survey

We used data from the 2020 Gallup World Poll, an annual cross-sectional survey administered to nationally representative samples of non-institutionalized individuals 15 years or older. Stratified sampling procedures were used to randomly select respondents, and probability sampling weights were generated to adjust for non-response and helped ensure estimates were representative of the civilian adult population in each country. Details on the sampling frame and methods used by Gallup World Poll to construct sampling weights are described elsewhere^30^^,^^31^ and in the online repository.^32^ In-country partners, who were trained on Gallup World Poll standardized guidelines for selecting and recruiting respondents and conducting interviews, administered the surveys.

### Measure of water insecurity

Water insecurity was assessed by Gallup using the Individual Water Insecurity Experiences Scale, which has been established as a reliable, cross-context equivalent and valid scale.^33^ The scale consists of 12 items about the frequency that individuals experienced life-disrupting water-related problems in the previous 12 months, including worrying about water, having to change what was eaten due to water problems, and having no water to drink (scale items are available in the online repository).^32^^,^^33^ Response options were never (scored as 0); 1 to 2 months (one point); some, not all, months (two points); or almost every month (three points). Scores were summed and the range could be 0 to 36. An individual was classified as water insecure if their score was ≥ 12.^33^

In-country partners in 31 low and middle-income countries administered the Individual Water Insecurity Experiences module of the 2020 Gallup World Poll. Details on the translation and implementation procedures used for administering the module have been published elsewhere.^33^ Briefly, the module was translated into major languages spoken in each country; experts reviewed and piloted the translated versions before implementation.^34^ Surveys were conducted by telephone in all but three countries due to coronavirus disease 2019 (COVID-19) restrictions; telephone sampling was used only if the combined landline and mobile telephone coverage in a country was ≥ 80%. In most countries, approximately 1000 individuals were sampled per country, with oversampling in China and India.

### Measure of food insecurity

The survey assessed food insecurity among the same individuals in 27 of the 31 countries using the Food Insecurity Experience Scale (online repository).^32^^,^^35^^,^^36^ The scale comprises eight items on whether individuals experienced constraints on food access resulting from lack of resources in the previous year, including worrying about food, having to reduce meal size and going for an entire day without eating (full list available in the online repository).^32^ Affirmative responses to the eight questions were scored as 1, otherwise 0, resulting in a maximum score of eight points.

The analytic protocol of Food Insecurity Experience Scale data is based on the Rasch model.^37^ To achieve cross-country equivalence of food insecurity estimates, we implemented the equating procedure to adjust for potential differential functioning of the scale in different countries (further details in the online repository).^32^^,^^35^ We categorized individuals as being in the moderate-to-severe food insecurity category if their individual Rasch-estimated equated probability parameter was ≥ 0.5. We also assessed the severity of food insecurity using the Rasch-estimated equated severity parameter, which represents a cross-country equivalent, quantitative, equal interval measure of food insecurity severity, expressed as a transformed logit metric.^36^

### Socioeconomic covariates

To account for potential confounding, we adjusted for socioeconomic factors that may be common causes of both water insecurity^19^^,^^38^^,^^39^ and food insecurity.^1^^,^^40^ These factors were per capita annual household income bracket, perceived adequacy of household income, current employment status, gender, age, urbanicity, household size, marital status, education level and COVID-19 pandemic-related life disruptions. We provide details about each of these factors in the online repository.^32^

### Statistical analysis

We conducted descriptive analyses and regression models using Stata, version 17 (StataCorp LLC, College Station, United States of America). We used Stata’s survey commands to account for sampling weights and regional stratification (online repository).^32^ We used the Gallup World Poll regions to categorize countries into sub-Saharan Africa, North Africa, Asia and Latin America. We combined South Asia and East Asia into one region because we only had data from three countries in Asia. For each country to contribute equally to models that pooled individuals across multiple countries, we normalized the sampling weights so that they summed to one for each country and thereby contributed equally to model results regardless of population or sample size. For descriptive statistics and estimating the proportions of adults who were water insecure and food insecure among the respondents, we used projection weights, generated by multiplying the normalized weights by World Bank 2020 estimates of the ≥ 15-year-old population in each country.^41^

To describe the extent to which water insecurity and food insecurity were concurrently experienced, we examined the proportion of individuals classified as water insecure who also experienced moderate-to-severe food insecurity during the same time. We then used logistic regression models to test how the odds of experiencing moderate-to-severe food insecurity varied by water insecurity status when adjusting for confounders in each country, region and the overall sample.

To understand how individuals’ severity of food insecurity covaried in relation to their severity of water insecurity, we built linear regression models regressing Rasch-estimated equated food insecurity severity parameters (expressed as a transformed logit metric) on Individual Water Insecurity Experiences score (modelled continuously) for each country, region and the overall sample.

To examine the degree to which socioeconomic factors confound the relationship between water insecurity and food insecurity, we compared unadjusted and fully adjusted coefficients for all logistic and linear regression models. To do this, we first built country models with no covariate adjustment and regional and overall sample models adjusted for only country fixed effects; we then added all socioeconomic covariates to those models.

## Results

Of the 27 countries surveyed with both the Individual Water Insecurity Experiences and Food Insecurity Experience modules, we had a base sample of 38 189 individuals. For individuals missing responses to one to three questions about water insecurity (3.3%; 1252 respondents), we imputed the data to calculate their water insecurity experiences score. We excluded 166 respondents who were missing data for more than three items of the Individual Water Insecurity Experiences module. We also excluded 611 respondents missing data for any item on the Food Insecurity Experience Scale. We excluded all 997 respondents from Zimbabwe due to unreliable income data; all 3468 respondents from China were excluded due to questions on COVID-19 pandemic-related life disruptions not being permitted in the China survey. Finally, 1241 respondents with missing or do not know responses to any of the covariate items were excluded (flowchart available in online repository).^32^ Our final analytic sample therefore comprised 31 755 individuals representing 25 countries and a population of approximately 1.65 billion individuals 15 years or older. Socioeconomic characteristics of the respondents are presented in Table 1.

**Table 1:** Characteristics of respondents of the 2020 Gallup World Poll on water and food insecurity in 25 low- and middle-income countries

Country by region (no. of respondents)	Age in years, mean (SD)	Female, %	Small town or rural resident, %	No. of household residents, mean (SD)		Marital status			Level of education			Work activity			Per capita household income, median Int$ (IQR)	Difficulty getting by on income, %		COVID-19 situation	
						Married or partnered, %	Separated, divorced or widowed, %		Secondary, %	Higher, %		Under-employed, %	Unemployed, no. (%)	Out of workforce, %				Life somewhat affected, %	Life greatly affected, %
**Sub-Saharan Africa**																			
Benin (959)	31.6 (12.8)	55.1	64.0	5.2 (2.7)		53.4	9.4		41.4	7.1		11.0	9.1	21.2	520 (173 to 1301)	68.7		30.0	43.4
Burkina Faso (966)	30.3 (11.0)	52.2	54.7	6.3 (3.9)		54.4	5.7		26.2	1.1		13.2	13.8	25.4	307 (99 to 878)	60.1		31.0	46.1
Congo (924)	36.1 (15.5)	51.9	41.9	5.8 (2.7)		41.6	13.8		66.8	2.1		10.6	15.9	30.0	435 (209 to 871)	72.2		33.6	28.5
Côte d'Ivoire (904)	31.0 (11.9)	48.0	37.6	5.7 (3.2)		45.4	6.3		37.7	1.9		11.2	10.3	27.9	577 (247 to 1483)	64.8		24.1	36.3
Gabon (952)	33.7 (13.2)	48.7	36.1	4.8 (2.7)		45.8	4.1		58.9	2.9		14.1	14.5	29.8	738 (111 to 1477)	66.7		32.8	47.8
Ghana (861)	31.6 (13.0)	47.8	45.1	6.6 (3.7)		39.5	6.9		70.7	7.7		10.3	11.7	25.1	543 (136 to 1272)	61.8		30.3	41.9
Guinea (939)	31.9 (13.7)	50.4	33.0	6.9 (4.3)		49.9	12.3		22.4	8.3		19.7	10.1	24.3	274 (25 to 739)	52.3		26.9	46.9
Kenya (982)	30.6 (11.3)	49.5	75.8	5.4 (3.0)		44.6	8.0		66.5	11.5		10.1	8.8	14.8	615 (224 to 1398)	63.3		30.1	61.5
Mali (907)	34.4 (15.8)	51.1	56.0	11.9 (6.9)		71.8	5.5		18.7	1.2		29.9	4.9	19.8	172 (37 to 430)	49.4		15.3	24.4
Mauritius (946)	42.0 (17.4)	45.2	71.3	4.0 (1.8)		58.1	11.8		55.1	10.2		6.1	5.0	29.4	3388 (2033 to 5647)	39.4		38.4	37.2
Namibia (929)	32.9 (13.1)	54.2	82.9	6.4 (3.8)		20.8	4.5		75.0	7.3		11.8	17.5	34.2	449 (140 to 1264)	78.1		19.6	61.1
Senegal (950)	34.4 (15.8)	53.7	62.4	10.7 (4.4)		57.7	7.6		23.5	0.8		12.6	11.3	45.2	532 (289 to 920)	59.4		26.5	51.4
Togo (963)	32.6 (13.2)	51.7	59.7	5.4 (2.5)		47.8	12.0		44.9	10.7		9.7	13.1	19.9	373 (166 to 663)	74.1		33.4	52.8
Uganda (948)	29.9 (10.2)	52.0	83.3	6.8 (3.9)		38.8	10.8		68.2	1.0		7.5	8.0	25.8	226 (33 to 573)	75.6		27.8	62.1
United Republic of Tanzania (973)	32.2 (12.4)	51.3	54.9	5.4 (2.8)		47.9	9.1		24.8	4.1		6.3	10.1	15.9	400 (167 to 999)	47.8		34.9	19.2
Zambia (982)	31.3 (13.1)	50.1	73.4	5.9 (3.0)		38.5	6.7		75.4	12.3		14.1	15.4	28.7	747 (235 to 1826)	74.9		23.8	57.6
Pooled subsample (15 085)	31.7 (12.7)	50.7	60.8	6.5 (4.1)		46.8	8.3		47.9	5.5		11.2	10.3	22.9	426 (137 to 1052)	61.8		28.9	44.5
**North Africa**																			
Algeria (1009)	36.3 (16.0)	47.7	29.6	4.6 (2.2)		48.2	8.7		53.2	11.2		3.9	9.9	52.2	2779 (993 to 5003)	25.9		48.3	37.0
Egypt (972)	35.4 (14.5)	47.0	50.6	4.7 (1.9)		62.0	6.6		35.3	16.0		6.1	9.6	46.2	1702 (1 064 to 2837)	46.6		26.4	58.1
Morocco (958)	37.5 (15.1)	50.7	42.8	5.2 (2.4)		53.7	9.5		30.9	6.7		7.9	12.2	50.3	1198 (340 to 2723)	33.2		38.0	46.9
Tunisia (954)	38.0 (16.3)	49.6	27.4	4.0 (1.9)		49.0	9.3		52.0	14.0		7.3	11.7	43.4	2839 (1 533 to 6387)	50.2		37.7	44.9
Pooled subsample (3893)	36.2 (15.1)	48.1	42.7	4.7 (2.1)		56.3	7.8		39.6	12.9		6.0	10.4	48.2	1773 (908 to 3404)	39.4		34.5	50.2
**Asia**																			
Bangladesh (951)	32.6 (12.3)	48.2	61.2	6.3 (2.7)		65.6	1.7		61.3	8.7		4.5	8.4	54.5	991 (413 to 1872)	31.4		37.6	50.1
India (8899)	36.2 (15.4)	48.2	77.1	4.8 (2.3)		66.5	5.9		26.4	5.8		10.6	11.9	40.3	816 (408 to 1632)	52.7		29.4	50.3
Pooled subsample (9850)	35.8 (15.2)	48.2	75.4	5.0 (2.4)		66.4	5.5		30.1	6.1		9.9	11.5	41.8	826 (408 to 1632)	50.4		30.3	50.2
**Latin America**																			
Brazil (971)	39.0 (17.2)	51.2	49.6	3.6 (1.8)		49.3	9.8		60.9	10.0		10.1	14.1	26.7	3331 (1499 to 6246)	31.6		43.8	41.5
Guatemala (1028)	35.0 (15.1)	50.3	80.1	5.9 (2.9)		52.4	8.9		53.7	3.8		9.6	9.5	27.7	891 (285 to 1872)	53.8		44.5	48.1
Honduras (928)	33.3 (14.5)	52.4	64.4	5.3 (2.5)		53.8	6.6		35.2	6.3		15.4	18.9	29.6	615 (231 to 1475)	73.0		27.8	52.1
Pooled subsample (2927)	38.6 (17.1)	51.1	51.9	3.8 (2.0)		49.7	9.6		59.5	9.5		10.2	14.0	26.9	2995 (1291 to 5746)	34.4		43.3	42.3

COVID-19: coronavirus disease 2019; Int$: international dollars; IQR: interquartile range; SD: standard deviation.

Note: All percentages are weighted.

**Fig. 1:**
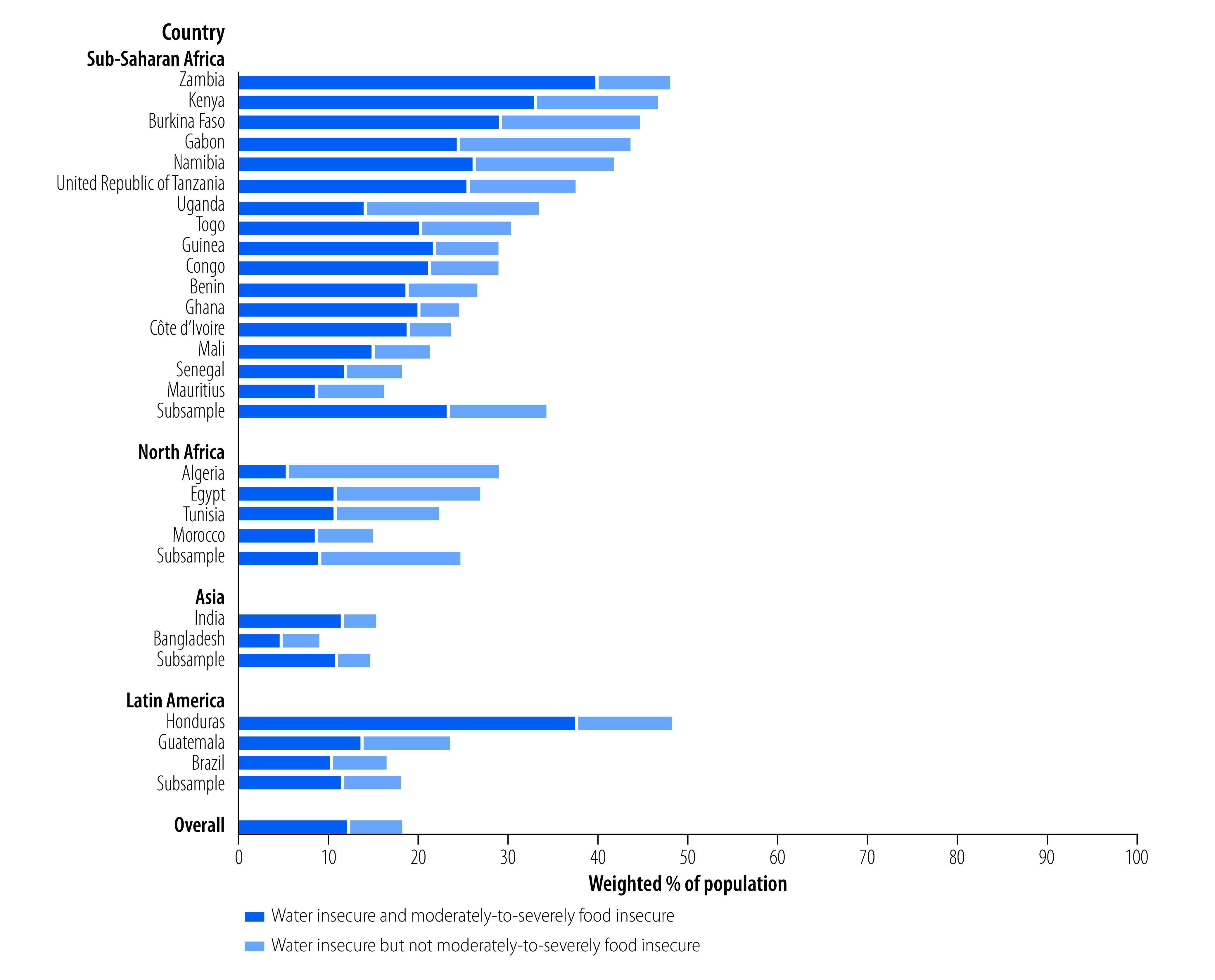
Individuals experiencing water insecurity and moderate-to-severe food insecurity, 25 low- and middle-income countries, 2020

Notes: The 2020 Gallup World Poll included 31 755 respondents. Estimates were weighted, using probability sampling weights for country estimates and projection weights for regional and overall pooled samples estimates. Food insecurity was defined as a Rasch-estimated equated probability parameter ≥ 0.5. Water insecurity was defined as an Individual Water Insecurity Experiences score of ≥ 12.

Of the 18.3% of respondents who experienced water insecurity in the preceding 12 months, more than half (66.8%) experienced moderate-to-severe food insecurity (Table 2; Fig. 1). Overall, 12.2% of respondents experienced concurrent water and food insecurity (Fig. 1). Concurrent water and food insecurities was highest in countries in Asia and sub-Saharan Africa, where 73.0% and 67.7% of the individuals who experienced water insecurity also experienced food insecurity, respectively (Table 2).

**Table 2:** Percentage of individuals experiencing water insecurity who also experienced moderate-to-severe food insecurity in the previous year, 25 low- and middle-income countries, 2020

Region and country	No. of people surveyed^a^	Weighted % of people	
		Experiencing water insecurity^b^	Experiencing water and food insecurity^b,c^
**Sub-Saharan Africa**			
Benin	959	26.6	70.1
Burkina Faso	966	44.8	65.0
Congo	924	29.1	72.7
Côte d'Ivoire	904	23.7	79.5
Gabon	952	43.7	55.9
Ghana	861	24.7	81.5
Guinea	939	29.1	74.7
Kenya	982	46.7	70.5
Mali	907	21.4	69.7
Mauritius	946	16.3	53.5
Namibia	929	41.9	62.3
Senegal	950	18.3	64.6
Togo	963	30.5	66.1
Uganda	948	33.5	41.9
United Republic of Tanzania	973	37.6	67.5
Zambia	982	48.2	82.4
Pooled subsample	15 085	34.4	67.7
**North Africa**			
Algeria	1 009	29.1	18.6
Egypt	972	27.0	39.2
Morocco	958	15.1	57.2
Tunisia	954	22.4	47.7
Pooled subsample	3 893	24.7	36.4
**Asia**			
Bangladesh	951	9.1	51.5
India	8 899	15.5	74.5
Pooled subsample	9 850	14.8	73.0
**Latin America**			
Brazil	971	16.6	61.9
Guatemala	1 028	23.6	57.7
Honduras	928	48.3	77.6
Pooled subsample	2 927	18.2	63.1
**Overall pooled sample**	**31 755**	**18.3**	**66.8**

^a^ People surveyed in the 2020 Gallup World Poll.

^b^ People with an Individual Water Insecurity Experiences score of ≥ 12.^33^

^c^ Food insecurity was defined as Rasch-estimated equated probability parameters of ≥ 0.5.

**Fig. 2:**
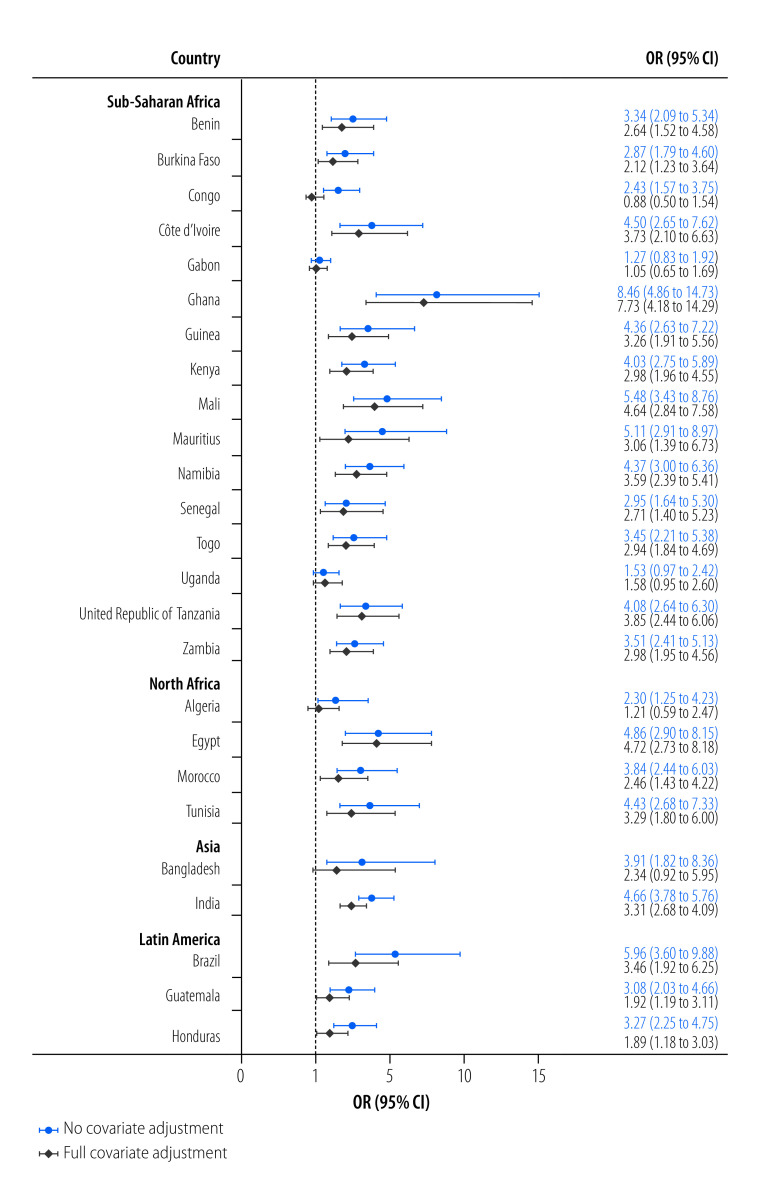
Unadjusted and adjusted odds of experiencing moderate-to-severe food insecurity in relation to water insecurity, 25 low- and middle-income countries, 2020

CI: confidence interval; OR: odds ratio.

Notes: The 2020 Gallup World Poll included 31 755 respondents. Food insecurity was defined as Rasch-estimated equated probability parameters ≥ 0.5. Water insecurity was defined as an Individual Water Insecurity Experiences score of ≥ 12. Models regressed food insecurity on water insecurity. Fully adjusted models control for income bracket, income bracket squared, difficulty getting by on income, age, gender, household size, urbanicity, marital status, education, employment status, and extent to which the coronavirus disease 2019 pandemic affected respondents’ lives (not at all, some, much).

**Fig. 3:**
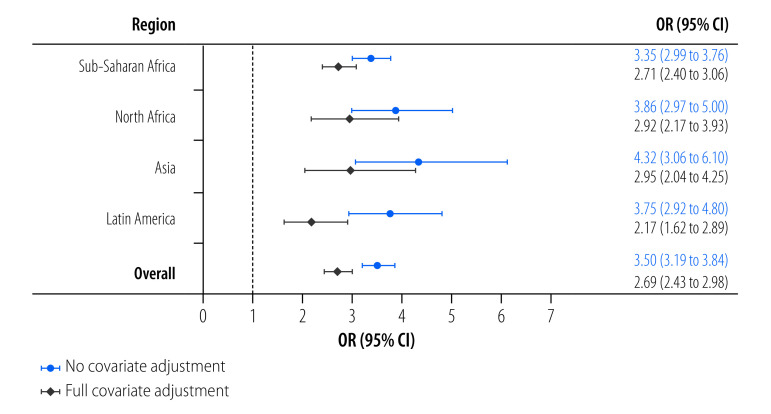
Unadjusted and adjusted odds of experiencing moderate-to-severe food insecurity in relation to water insecurity by region, 2020

CI: confidence interval; OR: odds ratio.

Notes: The 2020 Gallup World Poll included 31 755 respondents. Food insecurity was defined as Rasch-estimated equated probability parameters ≥ 0.5. Water insecurity was defined as an Individual Water Insecurity Experiences score of ≥ 12. Models regressed food insecurity on water insecurity, adjusting for country fixed effects. Fully adjusted models control for income bracket, income bracket squared, difficulty getting by on income, age, gender, household size, urbanicity, marital status, education, employment status, and extent to which the coronavirus disease 2019 pandemic affected respondents’ lives (not at all, some, much).

In the unadjusted model, the likelihood of experiencing moderate-to-severe food insecurity was higher when also experiencing water insecurity in most countries except for Gabon and Uganda, where no relationships were observed (Fig. 2). Positive associations between the two insecurities were also observed in all four regions in this model (Fig. 3). In the adjusted model, the relationships between the two insecurities were slightly attenuated but remained significant in all regions and most countries except for Algeria, Bangladesh, Congo, Gabon and Uganda (Fig. 2). Individuals experiencing water insecurity were more than twice as likely to experience moderate-to-severe food insecurity in Latin America (adjusted odds ratio, aOR: 2.17; 95% confidence interval, CI: 1.62 to 2.89) and nearly three times as likely in Asia (aOR: 2.95; 95% CI: 2.04 to 4.25), North Africa (aOR: 2.92; 95% CI: 2.17 to 3.93) and sub-Saharan Africa (aOR: 2.71; 95% CI: 2.40 to 3.06; Fig. 3).

**Fig. 4:**
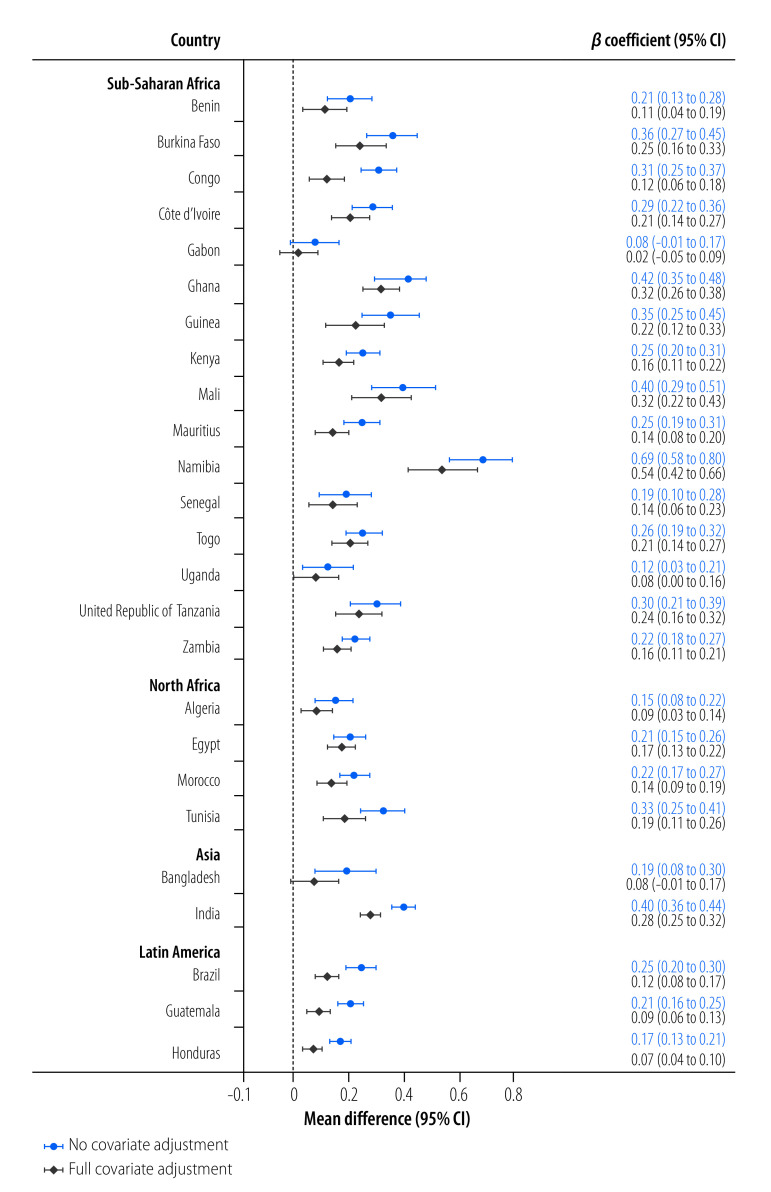
Severity of food insecurity in relation to water insecurity, 25 low- and middle-income countries, 2020

**Fig. 5:**
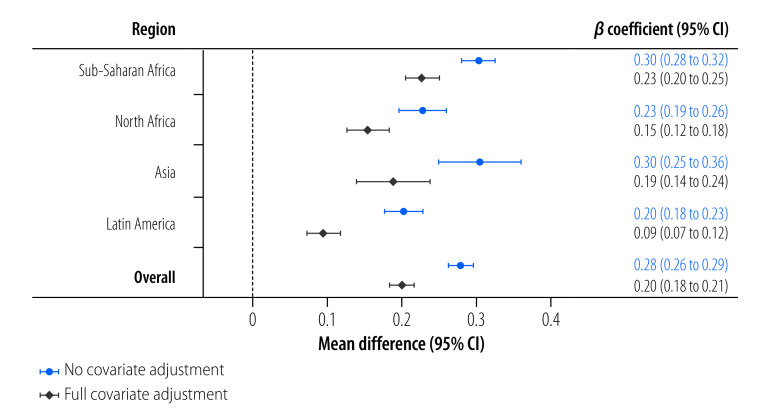
Severity of food insecurity in relation to water insecurity by region, 2020

The severity of food insecurity was positively associated with water insecurity severity in adjusted models in all but two countries, Bangladesh and Gabon (Fig. 4). This positive relationship was also strong in all pooled regional samples, even after adjusting for covariates (Fig. 5). In adjusted models for the overall pooled sample, every three points higher on the Individual Water Insecurity Experiences Scale that an individual scored was associated with a 0.20 (95% CI: 0.18 to 0.21) difference in the transformed logit food insecurity severity metric.

CI: confidence interval.

Notes: The 2020 Gallup World Poll included 31 755 respondents. Models regressed the Rasch-estimated equated severity parameters for Food Insecurity Experience scores (expressed as a transformed logit metric) on a three-unit difference in the raw Individual Water Insecurity Experiences score. Fully adjusted models control for income bracket, income bracket squared, difficulty getting by on income, age, gender, household size, urbanicity, marital status, education, employment status, and extent to which the coronavirus disease 2019 pandemic affected respondents’ lives (not at all, some, much).

CI: confidence interval.

Notes: The 2020 Gallup World Poll included 31 755 respondents. Models regressed the Rasch-estimated equated severity parameters for Food Insecurity Experience scores (expressed as a transformed logit metric) on a three-unit difference in the raw Individual Water Insecurity Experiences score. Fully adjusted models control for income bracket, income bracket squared, difficulty getting by on income, age, gender, household size, urbanicity, marital status, education, employment status, and extent to which the coronavirus disease 2019 pandemic affected respondents’ lives (not at all, some, much).

## Discussion

The concurrence of water and food insecurities was common in nationally representative samples from 25 low- and middle-income countries. In most countries and in all regions, these two forms of resource insecurities were strongly associated; this association was independent of income, financial difficulty, personal impact of the COVID-19 pandemic and demographic factors.

These results at the individual level corroborate findings of smaller, non-nationally representative studies. For example, a few studies demonstrated positive linear relationships between household food insecurity scores and the Household Water Insecurity Experiences Scale^9^^,^^19^^,^^42^ or household water access and quality measures^43^ when adjusting for proxies of socioeconomic status (e.g. perceived social status,^9^^,^^42^ water expenditures^19^ and land or livestock ownership).^43^ Our results are also consistent with the relationships reported between food insecurity and various measures of water scarcity, access or insecurity among populations that were already facing several resource- and health-related disadvantages such as smallholder farmers in Nicaragua,^18^ women in Cameroon,^21^^,^^24^ mothers affected by human immunodeficiency virus (HIV) in Kenya,^22^ smallholder farmers living with HIV in western Kenya,^22^ and men and women living in Lesotho, where HIV is endemic.^14^ This study adds to this literature by demonstrating in nationally representative samples that water insecurity is strongly associated with food insecurity independently of socioeconomic factors among individuals in the general population.

These findings have important public health implications in light of theories and evidence that water insecurity and food insecurity may have mutually exacerbating effects on physiological and mental health.^4^^,^^5^^,^^14^^,^^15^^,^^22^^,^^26^^,^^29^^,^^43^ For example, water insecurity or reduced water access and food insecurity have been independently associated with measures of psychological distress (e.g. depression and/or anxiety) among adults in Ethiopia,^44^ Haiti,^43^ Kenya,^5^^,^^22^ Lesotho^14^ and slums in India;^15^ worse physical health ratings among adults in Kenya;^5^ and elevated systolic blood pressure among women in Nepal.^26^ Not only may water and food insecurities exacerbate each other, they may also have additive – and potentially multiplicative – adverse effects on health when experienced concurrently.

Future research should examine the paths through which water insecurity affects food insecurity, nutritional intake and other food-related behaviours across different settings, and whether relationships are causal. The role that food insecurity may play in exacerbating water insecurity should also be examined. The relationships between water and food insecurities likely differ depending on food production and preparation strategies, climate and climate-change-related alterations in precipitation patterns, infrastructure and other conditions. Elucidating these paths will help explain why water insecurity and food insecurity were occasionally unrelated. Investigation of these relationships in additional low- and middle-income countries, as well as high-income countries, will extend understanding of the extent to which water and food insecurities are experienced concurrently around the globe.

Strengths of this study include the use of the first cross-context equivalent scale for a more holistic measurement of water insecurity than is permitted by other global indicators such as water stress or water infrastructure. This study also concurrently measured experiences of water and food insecurity among the same individuals in nationally representative samples. The variety of countries included, spanning four regions, and the large sample size are additional strengths.

Our study has some limitations. First, because the data we used are cross-sectional, we cannot determine causality or rule out the possibility that the relationship is bidirectional. Second, other unmeasured material-need insecurities and/or effects of the COVID-19 pandemic on availability and acquisition of resources could confound the results. There may be bidirectional relationships between impacts of the pandemic and water and food insecurities. Future analyses should evaluate if the relationship between water and food insecurity vary by individual characteristics, including sex and age; in the current analyses, we only controlled for these characteristics. Finally, the survey did not collect data on factors that might explain why the relationship between water insecurity and food insecurity was stronger in some countries than in others, such as respondents’ diet, water source and climate-related factors.

Here we provide evidence that water and food need to be considered together, an opinion expressed in a 2020 report by the United Nations (UN) Standing Committee on Nutrition^45^ and elsewhere.^6^^,^^7^ While the UN General Assembly designated 2018‒2028 as the UN International Decade for Action on Water for Sustainable Development^46^ and 2016‒2025 the UN Decade of Action on Nutrition to support the sustainable development goals,^47^ collaboration between these two commitments has been minimal.^45^ Although there is growing attention to the importance of water for food production, especially in regard to changing precipitation patterns due to climate change, the many other paths by which water insecurity may exacerbate food insecurity and nutrition have not been studied. These paths include the role that water insecurity plays in impeding food purchases, washing vegetables and fruits, meal preparation, and hydration during lactation. This gap in research is a lost opportunity for improving public health.

Although international, governmental and nongovernmental agencies and institutions have different responsibilities, priorities and expertise, the interdependencies of food and water insecurities could help to spur coordination between these entities to ensure that both are considered together where appropriate. Furthermore, the identical recall periods used at the individual level for the Food Insecurity Experience Scale and Individual Water Insecurity Experiences Scale may make it easier to bridge sectors using common measures and indicators. Evidence that improving one type of resource insecurity requires amelioration of the other would further motivate collaboration. For example, failure to consider the role of water issues in food insecurity could lead to less effective policies and interventions; provisioning of food without ensuring access to water to prepare that food could be an incomplete solution. Hence, our findings highlight the importance of measuring, monitoring and addressing water insecurity alongside food insecurity and other nutrition outcomes. 
